# Exploring the Role of Fallopian Ciliated Cells in the Pathogenesis of High-Grade Serous Ovarian Cancer

**DOI:** 10.3390/ijms19092512

**Published:** 2018-08-24

**Authors:** Michela Coan, Gian Luca Rampioni Vinciguerra, Laura Cesaratto, Emanuela Gardenal, Riccardo Bianchet, Erik Dassi, Andrea Vecchione, Gustavo Baldassarre, Riccardo Spizzo, Milena Sabrina Nicoloso

**Affiliations:** 1 Division of Molecular Oncology, Department of Translational Research, IRCCS CRO Aviano-National Cancer Institute, Via Franco Gallini, 2 33081 Aviano PN, Italy; michela.coan@gmail.com (M.C.); Gianluca.rampionivinciguerra@uniroma1.it (G.L.R.V.); lacesaratto@gmail.com (L.C.); gbaldassarre@cro.it (G.B.); mnicoloso@cro.it (M.S.N.); 2 Azienda Ospedaliera Universitaria Integrata, University of Verona, 37129 Verona, Italy; emanuela.gardenal@gmail.com; 3 Scientific Direction, CRO Aviano Italy, Via Franco Gallini, 2 33081 Aviano, Italy; rbianchet@cro.it; 4 Centre for Integrative Biology, University of Trento, 38122 Trento, Italy; erik.dassi@unitn.it; 5 Department of clinical and molecular medicine, university of Rome “Sapienza”, c/o sant andrea hospital, Via di Grottarossa 1035, 00189 Rome, Italy; andrea.vecchione@gmail.com

**Keywords:** epithelial ovarian cancer, predisposition, ciliated cells, *CCDC170*, *DNAAF1*, *LRRC46*, *MARCH10*, *C20orf85*, *LRP2BP*, *SPAG6*, *TPPP*, *RSPH10B2*, *STK33*

## Abstract

High-grade serous epithelial ovarian cancer (HGSOC) is the fifth leading cause of cancer death in women and the first among gynecological malignancies. Despite an initial response to standard chemotherapy, most HGSOC patients relapse. To improve treatment options, we must continue investigating tumor biology. Tumor characteristics (e.g., risk factors and epidemiology) are valuable clues to accomplish this task. The two most frequent risk factors for HGSOC are the lifetime number of ovulations, which is associated with increased oxidative stress in the pelvic area caused by ovulation fluid, and a positive family history due to genetic factors. In the attempt to identify novel genetic factors (i.e., genes) associated with HGSOC, we observed that several genes in linkage with HGSOC are expressed in the ciliated cells of the fallopian tube. This finding made us hypothesize that ciliated cells, despite not being the cell of origin for HGSOC, may take part in HGSOC tumor initiation. Specifically, malfunction of the ciliary beat impairs the laminar fluid flow above the fallopian tube epithelia, thus likely reducing the clearance of oxidative stress caused by follicular fluid. Herein, we review the up-to-date findings dealing with HGSOC predisposition with the hypothesis that fallopian ciliated cells take part in HGSOC onset. Finally, we review the up-to-date literature concerning genes that are located in genomic loci associated with epithelial ovarian cancer (EOC) predisposition that are expressed by the fallopian ciliated cells.

## 1. Introduction

Ovarian cancers include three main types—epithelial ovarian cancers (EOC), sex-cord stromal tumors, and germ cell tumors—with EOC being the most frequent and lethal among them. Worldwide, EOC has an estimated age-standardized rate (ASR) of six new cases per year per 100,000 persons [1], which, according to European Commission guidelines, makes EOC a rare tumor [2]. EOC is more frequent in developed regions, and Europe has the highest ASR, particularly in the Central and Eastern Europe regions (e.g., Belarus, Bulgaria, etc.) with up to 14 new cases per year per 100,000 persons in Bulgaria [1]. At the same time, encouraging data from the Surveillance, Epidemiology, and End Results (SEER) Program reported a 1.1% observed annual reduction in EOC incidence in the United States between 1991 and 2015 [3].

Despite its low incidence, EOC is the fifth leading cause of cancer death in women and the first among gynecological tumors in western countries [1]. The high mortality rate of EOC is due to advanced stage at diagnosis (i.e., when the tumor has already spread outside the pelvic area) and to the development of resistance to standard platinum/taxane chemotherapy.

To improve EOC patients’ clinical outcome, we must diagnose EOC at earlier stages, and stratify patients according to best treatment options and to identify novel therapeutic targets. For all these reasons, it is crucial to continue investigating EOC tumor biology and clinical characteristics (e.g., risk factors, clinic, and epidemiology), which contain valuable information to accomplish this task [4].

## 2. Epithelial Ovarian Cancers: Classification and Cell of Origin

EOC is a complex disease; it includes four major histotypes (i.e., serous, endometrioid, mucinous, and clear cell), which are further classified as low grade (well differentiated) or high grade (poorly differentiated) based on cytological atypia. In addition to its histopathology traits, EOC is heterogonous due to genetic and clinical characteristics, for which EOC is divided into two main types (i.e., I and II) (for a through review on this topic, please refer to References [5,6]). Briefly, type I tumors are less frequent and include low-grade serous and endometrioid, clear-cell, mucinous carcinomas and Brenner tumors. They are genetically stable, tend to be clinically indolent, and are usually diagnosed at early stages; although, when diagnosed at advanced stages, type I tumors tend to have a poor outcome. Type II tumors are more frequent and include high-grade EOC (primarily high-grade serous ovarian cancer), undifferentiated, and malignant-mixed mesodermal tumors. They typically present at advanced clinical stage, and exhibit high chromosomal instability with more than 80% displaying *TP53* mutations and alterations of the homologous recombination DNA repair pathway [7]. Endometrioid cancers are about 10% of all EOCs; they are typically diagnosed at early stage and are low-grade tumors [8]. Similarly to colorectal and gastric cancers, an increased risk of developing endometrial cancer is associated with Lynch syndrome, a condition caused by germ-line pathogenic variants in the highly penetrant mismatch repair genes, *MLH1*, *MSH2*, *MSH6*, and *PMS2* [9]. Clear-cell carcinomas account for 5% of EOCs, and they are more frequent in the Japanese population [10]. Clear-cell cancers frequently develop chemoresistance with a worse patient outcome in advanced stages compared with serous EOCs. Both endometrioid and clear-cell tumors are strongly associated with endometriosis, and they show frequently inactivating mutations of the *ARID1A* gene [10]. Mucinous cancers account for about 10% of EOCs, they are characterized by the mutation of *KRAS*, whereas *BRCA1/BRCA2* and *TP53* are typically not mutated, which suggests that they develop along a separate pathway [11]. Low-grade serous EOCs (LGSOCs; <5% of EOCs) typically arise at younger ages and have mild-to-moderate cytological atypia and a low mitotic rate. LGSOCs tend to have a better survival than high-grade serous EOCs (HGSOCs), even though LGSOCs do not respond to traditional chemotherapy in the advanced stages [12,13].

High-grade serous ovarian cancers (HGSOC) are the single most frequent EOC histotype (about 70–80% of all EOCs) and account for the majority of EOC deaths. They are typically diagnosed when the primary mass is large, invades several pelvic organs, and/or disseminates to the peritoneum; thus, it is difficult to understand the precise anatomic site of HGSOC origin. For a long time, HGSOCs were thought to originate from the surface epithelium of the ovary; however, a decade ago, studies on fallopian tube specimens from prophylactic salpingo-oophorectomy in *BRCA*-mutation carriers generated a quite compelling shift on the field of HGSOC origin [14,15,16,17,18]. These studies reported the existence of tubal lesions resembling the histology and genetic features of HGSOCs, and thus, they created new pathological entities (e.g., “serous tubal intraepithelial lesions” (STILs), “tubal intraepithelial lesions in transition” (TILT), “secretory cell outgrowths” (SCOUTs), and “p53 signatures”), which are now hypothesized to be the precursor lesions of HGSOC. This hypothesis shifted the attention from the ovarian surface epithelium, which derives from the coelomic epithelium, to the female genital epithelium, which instead derives from the Müllerian duct [19,20,21]. In line with this evidence, in the last decade, serous carcinomas of the ovary, of the fallopian tube, and of the peritoneum were all grouped together as a single entity (i.e., pelvic serous disease), which originates either from the serous epithelial cells of the fallopian tubes or from the secondary implants of the Müllerian duct, which are commonly localized on the ovary or on the peritoneum [4,22]. Intriguingly, the stem cells that periodically repopulate epithelial cells lining the endometrium and the fallopian tube reside at the fimbria where the Müllerian and the coelomatic epithelia merge, and where most STIL lesions occur [23,24,25]. In conclusion, EOCs are a very diversified group of tumors and remain without a defined cell of origin.

## 3. HGSOC Predisposition

Both environmental and genetic risk factors predispose one to EOCs. Interestingly, in the last twenty years, the intense study of EOC genetic risk factors offered valuable clues to meliorate EOC management. In 1994 and 1995, *BRCA1* and *2* genes were associated with familial and early-onset cases of breast and ovarian cancer, respectively [26,27,28]. *BRCA1* and *2* mutations not only increase the risk of developing EOCs, but they also impact on EOC progression. Indeed, EOC patients carrying germ-line *BRCA* mutations have a 98% response rate (complete and partial) to first-line platinum-based chemotherapy regimens versus 60% in nonhereditary controls; this favorable response rate persists also in the second and third platinum-based line treatments, which altogether explains the better overall survival of *BRCA*-mutation carriers compared with wild-type EOC patients (median survival 8.4 years versus 2.9 years) [29].

*BRCA1* and *2* genes are part of the DNA damage repair pathway regulating the homologous recombination (HR) mechanisms [30,31,32]. Later publications showed that characteristics of the deficient base excision repair pathway, such as deficiency of the poly-ADP-ribose polymerase (PARP1) enzyme, increased HR activity, and contrarily, that HR-deficient cells (e.g., due to *BRCA1* and *2* mutations) were hypersensitive to PARP1 inhibition [33,34]. These findings were later translated into clinical trials in platinum-sensitive relapsed patients that demonstrated significant benefits with *PARP1*-inhibitor (PARPi) treatment compared with a placebo, especially in EOC germ-line *BRCA*-mutation carriers (19–21 versus five months of progression-free survival) [35,36]. These evidences guaranteed approval by national drug administrations of PARPi as maintenance therapy after relapse to platinum-based chemotherapy in platinum-sensitive EOC germ-line *BRCA*-mutation carriers.

The example of *BRCA1* and *2* demonstrates that investigating the biological mechanisms of EOC risk factors can unveil new EOC Achilles’ heels, and eventually, suggest novel therapeutic approaches. Environmental risk factors with adequate evidence (based on study design, internal and external validity, and consistency among studies) are the lifetime number of ovulations, tubal ligation (30% relative risk reduction), breast-feeding (2% relative risk reduction for every month of breast feeding) [37], high body mass index (BMI; 7% relative risk increase per five-unit increase) [38,39], and endometriosis (80% to 140% relative risk increase) [40,41,42]. Factors with inadequate evidence (based on inconsistency of data or poor study design) are diet (e.g., alcohol consumption), smoking, perineal talc exposure, and the use of aspirin and of other nonsteroidal anti-inflammatory drugs [8,43].

Among environmental risk factors, the one resulting in the highest risk of EOC is the number of lifetime ovulations, which positively correlates with increased risk [44]. On the contrary, the use of oral contraceptives (OC) and parity, which both stop ovulation, proportionally decrease the risk of developing HGSOCs. For instance, the Collaborative Group on Epidemiological Studies of Ovarian Cancer showed that consistent OC users have a relative risk of 0.73 (95% confidence intervals (CIs): 0.7–0.76) compared to OC non-users, and that long-time users (15 years or more) have a 50% reduction in the risk of developing EOC compared to non-users [45]. These epidemiologic evidences were also confirmed by in vivo experiments using rat models, in which extra cycles of ovulation increased the incidence of preneoplastic lesions [46]. Similarly, in the modern egg-layer industry, hens ovulate and lay eggs daily, and they develop ovarian cancer in 35% of cases by 3.5 years of age [47]. In this model, reducing the number of ovulations (e.g., by using OCs or decreasing calorie uptake) reduced cancer incidence from 25% to 6% [47].

The biological mechanisms explaining the positive association between lifetime number of ovulations and EOC incidence are still a matter of debate. In 1971, Fathalla first proposed the “incessant ovulation” hypothesis [48], which stated that ovulation damages ovary surface epithelia and generates a scar, and that the repeated damage/repair cycles over time represents the soil for tumor development. However, this hypothesis was “coelomic-centric” and does not comply with the current Müllerian-centric cell of origin of HGSOCs [15]. A second hypothesis to explain the correlation between lifetime number of ovulations and HGSOC risk is based on the repeated hormonal stimulus of fallopian epithelia during the normal menstrual cycle, which may explain the positive correlation between replacement hormonal therapy during menopause and HGSOC risk [4]. A third hypothesis is that ovulation releases inflammatory molecules (e.g., prostaglandins) that recruit inflammatory cells, and thus, ovulation generates oxidative stress and genotoxic damage in the fallopian tube epithelia, which, repeated over time, predisposes one to tumor onset [49]. King et al. explored these three hypotheses in vivo (using a CD1 mouse model) and in vitro (using primary fallopian cell cultures), and they reported that ovulation or exogenous hormone stimuli did not induce fallopian epithelial cell proliferation. On the contrary, ovulation increased macrophage infiltration in the oviduct with an increased number of DNA-damaged cells [50]. It is worth mentioning that the mouse oviduct was not responsive to estrogen stimulus, as shown by King et al. [50]; however, the hen oviduct and human fallopian tube epithelia are responsive to estrogen [47,51].

The second most prevalent risk factor for EOC is a positive family history: females with a single first-degree relative affected by EOC have a three-fold increase in the risk of developing EOC compared with women in the general population [52]. Familial aggregation of EOC can be explained either by genetic or environmental factors (e.g., exposure to specific habits or pollutants) [53]; however, studies comparing mono and dizygotic twins in the northern populations suggest that genetic factors prevail for EOC. Monozygotic twins whose co-twin developed EOC had a three-fold greater risk of developing EOC as compared to dizygotic twins [53].

Genetic factors associated with HGSOC predisposition can either be rare in the general population with high–moderate penetrance (e.g., *BRCA1* and *2* mutations, and other mutations in DNA repair and mismatch repair genes) or common, but with low penetrance. High–moderate penetrant genetic factors explain about 20% of the excess of familial risk, with *BRCA1* and *2* mutations being the major contributors, whereas a polygenic model with multiple low-penetrant genetic variants explains the remaining 80% of familial risk [43]. Genome-wide association studies (GWASs) identified several low-penetrant genetic variants (e.g., single-nucleotide polymorphisms—SNPs) associated with increased EOC risk [54]. Many of these genetic variants are located in non-coding intergenic regions of the human genome, and are likely impacting the transcription of neighboring transcripts [55]. The most significant SNP associated with EOC occurrence is rs3814113, which is located in an intergenic region of the short arm of human chromosome 9 (9p22.2) [56], and whose closest gene is basonuclin2 (*BNC2*), which impacts on cell survival of EOC cell lines upon oxidative stress [57]. GWASs pinpointed many other genomic regions associated with EOC that still need to be investigated and that can harbor novel genes responsible for EOC predisposition and those involved in EOC biology [52,57,58].

## 4. Novel Candidate Genes Associated with HGSOC Predisposition

To expand the list of candidate genes associated with EOC predisposition, we used SNPs that were previously associated with EOC onset as marks of genomic intervals containing transcripts potentially involved in EOC predisposition [57,58]. We downloaded all SNPs associated with EOC from the European Bioinformatics Institute GWAS Catalog [54], which were previously published and reported to be significantly associated with EOC according to *p*-values. We also included the statistically significant SNPs published in the meta-analysis by Reference [58]. By these means, we found 76 SNPs associated with EOC: 70 are nucleotide substitutions and six are indels (Supplementary Table S1 and Supplementary Material GWAS_SNPs.bed). In order to define genomic intervals around the 76 SNPs, we arbitrarily set a 2-Mb interval (1 Mb upstream and 1 Mb downstream for each of the 76 SNPs). We also looked at other SNPs in linkage disequilibrium (*r*^2^ ≥ 0.5) with the 76 SNPs in the European population according to Reference [59], and all of them were included within the 2-Mb interval. If two or more of the 76 candidate SNPs were less than 1 Mb apart from each other, we grouped them in the same interval. Eventually, we obtained 54 genomic spans, which contain the 76 SNPs associated with EOC (Supplementary Table S1).

To select potential candidate genes that are within the 54 genomic spans and that are associated with EOC, we hypothesized that the most likely candidate genes should be differentially expressed between normal fallopian tube epithelia (FTE) and EOC samples. Therefore, we used two independent published gene expression datasets (GSE69428 and GSE10971) from the Gene Expression Omnibus Database [60] that report the gene expression profile of micro-dissected non-malignant FTE and HGSOC samples using the Affymetrix Human Genome U133 Plus 2.0 Array platform. GSE69428 compared the expression of 10 non-malignant FTE versus 10 HGSOC samples [61]; whereas, GSE10971 compared 24 non-malignant FTE (carrying or not germ-line mutations in *BRCA1/2* genes) versus 11 HGSOC samples (either *BRCA1/2* wild-type or mutant) [62]. Once we identified all significant probe sets (adj_*p*-value < 0.05 and logFC > |1|) comparing FTE versus HGSOC samples and concordant between GSE69428 and GSE10971 (Supplementary Material RStudio_analysis.txt, GSE10971_degs_all.txt and GSE69428_degs_all.txt), we selected those located within the 54 genomic regions associated with EOC predisposition (Supplementary Table S2). By these means, we eventually identified 141 probe sets (i.e., 141 genes) (Supplementary Table S3).

Among the 141 candidate genes, which are differentially expressed between FTE and HGSOC samples and located near SNPs associated with EOC predisposition, we focused on those genes that are expressed mostly in the cervix, endometrium, fallopian tube, or ovary tissues, thus suggesting a female genital-tract-specific function. To do this, we interrogated two publically available databases, the Genotype-Tissue Expression (GTEx) [55] and the Human Protein Atlas (HPA) [63] initiatives, which aim to profile the expression of all human genes and proteins in the majority of human tissues. Firstly, we selected 25 normal tissues that were profiled both by GTEx and HPA and that included the cervix, endometrium, fallopian tube, or ovary tissues. Next, for these 25 tissues, we retrieved RNA sequencing (RNA-seq) expression values of the 141 candidate genes from GTEx and HPA. Secondly, for each of the 141 genes, we ranked the 25 tissues from greatest to lowest gene expression both for the GTEx and HPA gene expression data. Eventually, we identified 28 genes for which at least one human female genital-tract tissue (cervix, endometrium, fallopian tube, or ovary) was ranked in the first or second position both in the GTEx and HPA datasets (Supplementary Tables S4–S6).

**Figure 1:**
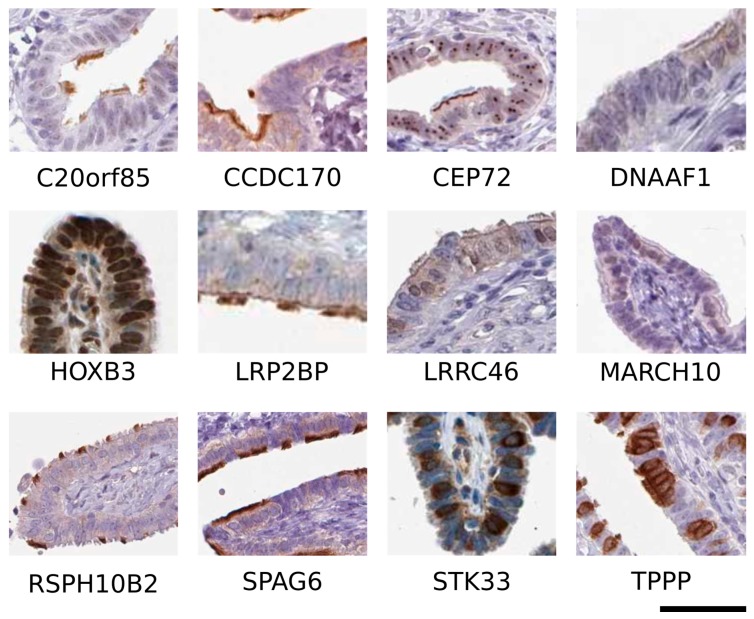
Representative images of immunohistochemistry staining of candidate genes from the Human Protein Atlas (HPA) database. Scale bar 50 μm.

Gene expression data alone cannot pinpoint the cellular/subcellular localization of genes in a tissue context. Therefore, for each of the 28 candidate genes, we looked at the immunohistochemistry (IHC) images publically available in the HPA dataset. The images were analyzed independently by two researchers, who, based on cytological features, identified ciliated cells (cuboid cells with cilia exposed on the apical surface) and serous cells (non-ciliated columnar cells, with darker nuclei and a granular cytoplasm containing secretory granules that are released within the lumen). Next, for each of the 28 genes, the two researchers independently described the cellular localization, and classified the protein localization as one of the following: localized in ciliated cells, localized in serous cells, localized in ciliated and serous cells, not expressed, or ambiguous (e.g., inconsistent between different tissue sections, or diffuse positivity both in the epithelium and in the connective tissue). Eventually, we compared the results of the two researchers. Discordant cases were discussed jointly, and eventually, a common decision was reached. Final descriptions are reported in Table 1, and examples of cell localization are shown in Figure 1.

For 11 genes, the antibodies showed an ambiguous localization pattern among different samples, while, for three genes, images were not yet available, and, for 1 gene, the IHC was completely negative. Interestingly, out of the 13 proteins that showed consistent and convincing expression, we noticed that 12 showed a specific positivity in the ciliated cells. The 12 proteins are localized in 10 different genomic intervals (Table 1), which ultimately means that 18% of the initial 54 genomic intervals associated with HGSOC (10/54) contain genes expressed in ciliated cells. Moreover, three out of 12 proteins expressed in ciliated cells are the most likely candidates to be associated with EOC risk. *C20orf85* and *SPAG6* were the only genes within their own genomic interval to be differentially expressed between FTE and HGSOC samples (Supplementary Table S3), and *STK33* was the closest gene to SNP rs16937956 (Supplementary Materials GWAS_SNPs.bed). Therefore, we hypothesized that ciliated cells may play a role in HGSOC initiation and predisposition.

## 5. Ciliated Cells in the Fallopian Tube: Function and Tumor Predisposition

Anatomically, the human fallopian tube is divided into the interstitial, isthmus, ampulla, and fimbria sections. Two major cell types compose the FTE: serous and ciliated cells. Serous cells are secretory by nature and produce the liquid film that overlays the epithelium; on the other hand, ciliated cells are specialized cells that contain motile cilia at their apical border. Ciliated cells are more abundant in the fimbria section (50%) and progressively decrease in number toward the uterus (30%), and they are mostly located at the apex of epithelial papillae [65,66]. Ciliated cells have hundreds of motile cilia with a length of ~10 µm and a diameter of 0.25 µm [67]. Motile cilia have a central axoneme made by nine peripheral microtubule doublets (MTDs) surrounding a central pair of microtubules (CP) (so-called 9 + 2 structure). Bridges of nexin and dynein complexes bind the nine MTDs to each other. In addition to being found in the FTE, ciliated cells are present in a few other anatomical districts in the human body (e.g., upper and lower airways, and ependymal cells of the brain ventricles) and in mature male germinal cells, which have a specialized type of cilia (i.e., the flagellum) [68]. Despite commonalities, protein composition differs among cilia from different epithelia [69].

The main function of ciliated cells in FTE is to transport the ovum from the ovary toward the uterus with a laminar fluid flow that Raidt, Werner, et al. estimated to be 32.43 µm/s [69]. In the follicular phase of the menstrual cycle, secretory cells increase their secretory activity, and, in the periovulatory phase, they reach their maximum height and secrete their content. After ovulation and following follicular and peritoneal fluid exposure, cilia increase their beat frequency and transport the ovum along the fallopian tube [66]. Indeed, pathologies that affect motile cilia activity (e.g., primary ciliary dyskinesia) are characterized by impaired fertility along with impaired airway mucociliary clearance [69]. Ciliary beat frequency (CBF) is regulated by several stimuli (e.g., calcium levels, adenosine triphosphate, angiotensin II, and β-adrenergic stimuli) [70,71]; high levels of estrogen in the pre-ovulatory phase increase CBF and ciliogenesis, whereas high levels of progesterone induce deciliation and decrease CBF [71,72,73,74,75,76,77]. Remarkably, follicular fluid generates a genotoxic stress in fallopian epithelial cells due to an oxidative burst after ovulation [50,57,78]; at the same time, CBF increases immediately after ovulation, as does the speed of laminal fluid flow above the FTE [76]. Therefore, it is very likely that ciliated cells not only favor the transit of the ovum through the fallopian tube toward the uterus, but they also provide a follicular fluid clearance, which removes the genotoxic stress after ovulation.

To our knowledge, there are no publications reporting that impaired cilia motility causes persistence of ovulatory genotoxic stress in the fallopian tube epithelium (FTE). At the same time, there are several indirect evidences that link ciliated cell function (i.e., CBF) or ciliogenesis to risk factors of EOC insurgence. For instance, endometriosis, which is a known risk factor of EOC onset [21,79,80], decreases fallopian CBF [66,81]. Similarly, HGSOC insurgence correlates with aging, which is also associated with a reduction in the number of ciliated epithelial cells. High-risk individuals (e.g., *BRCA* mutations, former breast cancer patients, and first degrees of ovarian cancer patients) and patients affected by pelvic serous carcinomas show, on average, a 50% reduction in the number of ciliated cells compared with women in the general population [65]. Ciliated cells and serous cells respond differently to DNA-damaging agents. Levanon et al. described an in vitro human fallopian tube model, which contained both ciliated and serous cells, and tested it with ionizing radiations, chemotherapy, or hydrogen peroxide. For all genetoxic stresses tested, less ciliated cells showed signs of DNA damage, which lasted less time compared with serous cells [82]. Finally, *TP53* mutations, which take part in EOC transformation [7], regulate ciliated cell differentiation as well [83]. This could be the case because ciliated and serous cells share common progenitors, but diverse differentiation stimuli (17β-estradiol for ciliated cells and progesterone for serous cells) [77]. George and Milea transduced a *TP53* gene carrying a missense mutation in the vitro FTE model of Levanon et al. [82], and observed that the mutant *TP53* prevented the differentiation of ciliated cells [83]. Similarly, the loss of *TP53* in airway progenitor cells prevents differentiation toward ciliated cell lineage [84].

To sum up, the findings that several genes present in genomic spans associated with EOC are expressed in FTE ciliated cells (Figure 1 and Table 1), and that several environmental and genetic factors involved in EOC onset affect cilia function suggest that FTE ciliated cells can be involved in EOC tumorigenesis.

## 6. Overview of Candidate Genes Expressed in Ciliated Cells

In this section, we review the current literature concerning the 12 genes that we identified as candidate genes associated with HGSOC predisposition, expressed in ciliated cells (Table 1 and Figure 1). As might be expected, most of these genes regulate tubulin and microtubule assembly or are a part of protein complexes that assemble motile cilia. Interestingly, three of them (*RSPH10B2*, *STK33*, and *TPPP*) are expressed only in some ciliated cells within the FTE (Table 1), which might suggest their involvement in ciliogenesis.

### 6.1. Chromosome 20 Open Reading Frame 85 (C20orf85)

According to the Human Protein Atlas, *C20orf85* is equally expressed in all anatomical sites containing ciliated cells. *C20orf85* (also known as low in lung cancer 1) was initially described to be downregulated in lung cancer samples compared to normal tissue [85]; subsequently, the same authors showed that *C20orf85* localizes to the ciliated cells of the upper airways, and, when overexpressed in cell models of lung cancer, did not affect proliferation or migration [86]. Within its own genomic interval, *C20orf85* is the only gene to be differentially expressed between FTE and HGSOC samples (Supplementary Table S3).

### 6.2. Coiled-Coil Domain-Containing Protein 170 (CCDC170)

According to the Human Protein Atlas, *CCDC170* is equally expressed in all anatomical sites containing ciliated cells. None of the 37 publications concerning *CCDC170* reports its function in motile cilia regulation; yet, *CCDC170* is associated with microtubule stabilization through α tubulin acetylation [87], which is also critical for motile cilia. *CCDC170* is only 133 kb away from estrogen receptor 1 (*ESR1*), and the closest SNPs to *CCDC170* are associated with breast cancer and endometriosis risk [88,89]. One possibility is that *ESR1* is responsible for the linkage between these SNPs and breast cancer or endometriosis. However, these SNPs are associated with *ESR1*-negative and *BRCA*-mutated breast tumors, suggesting an *ESR1*-independent linkage and pointing at *CCDC170* instead [89]. Indeed, overexpression of the C-terminal truncated CCDC170 protein, which originates from a translocation between *ESR1* and *CCDC170*, increased the migration ability of tumor cells [90].

### 6.3. Centrosomal Protein 72 (CEP72)

According to the Human Protein Atlas, *CEP72* is highly expressed in the testis, whereas it shows a three-fold less intense expression in all other tissues. CEP72 is a centriolar satellite protein, which interacts with other proteins associated with the centrosome, and it promotes centriole duplication during mitosis [91]. It is responsible for organizing microtubule activity and for the formation of the bipolar spindle [92]. CEP72 is also involved in ciliogenesis allowing the delocalization of Bardet–Biedl syndrome (BBS) proteins from the centriole to the primary cilium; indeed, the loss of centriolar satellites in zebrafish was demonstrated to cause cilium dysfunction, similarly to human ciliopathies [93]. CEP72 interacts with CEP290, which is a centrosomal protein that also localizes in the nucleus, and it regulates CEP290 localization to the centriolar satellites. Overexpression of *CEP72* (e.g., in HGSOC; see Table 1) sequesters CEP290 to aggregates, prevents primary cilium formation, and ultimately mimics the loss of CEP290 [93], which causes supernumerary centriole DNA damage due to a reduced replication fork velocity, fork asymmetry, and increased levels of cyclin-dependent kinases [94]. The CEP72 protein is associated with several cancers: it is upregulated in osteosarcoma [95], and a meta-analysis study identified a risk locus for Barrett’s esophagus and esophageal adenocarcinoma near the *CEP72* gene [96]. In addition, an increase in *CEP72* gene dosage was found in early stages of non-small cell lung cancer, and it might be used as a biomarker of detection and classification of lung cancer [97]. Interestingly, in colorectal cancer models, the CEP72 protein interacts with BRCA1 during mitosis, and its overexpression decreases *BRCA1* expression and induces chromosomal instability [98], which could also explain its putative relevance in EOC.

### 6.4. Dynein Axonemal Assembly Factor 1 (DNAAF1)

According to the Human Protein Atlas, *DNAAF1* is expressed in the female and male genital tract and at least 10-fold less in the brain and airways. DNAAF1 is the prototype of the motile cilia protein because it takes part in the assembly and stability of the outer and inner dynein arm of motile cilia [99]; mutations of *DNAAF1* cause primary ciliary dyskinesia-13. *DNAAF1* was found to be the most frequent gene carrying disruptive mutations in 153 independent European families affected by testicular germ-cell tumors (TGCT). In these families, the *DNAAF1* mutation was monoallelic, and in two of them, researchers demonstrated that tumors showed inactivation of the wild-type allele [100]. In a zebrafish model, the monoallelic disruption of *DNAAF1* generated TGCT in 94% of instances compared to 14% in the wild-type fish.

### 6.5. Homeobox B3 (HOXB3)

According to the Human Protein Atlas, *HOXB3* is mostly expressed at high levels in the male and female genital tract. *HOXB3* encodes a nuclear protein with a homeobox DNA-binding domain with transcription factor activity. It controls the positioning of cells in the anterior/posterior axis, and regulates angiogenesis [101,102] and the proliferation and differentiation of hematopoietic cells [103,104,105,106,107,108,109]. There are no publications reporting a direct role of *HOXB3* in ciliogenesis.

*HOXB3* was implicated in several tumors, such as acute myeloid leukemia [110,111,112,113,114,115,116,117], acute lymphoblastic leukemia [118], breast cancer [119,120,121], lung adenocarcinoma [119,122], oral squamous cell carcinoma models [123], gastric cancer [124], pancreatic cancer [125,126], osteosarcoma [119], and glioblastoma [127]. *HOXB3* gene overexpression was also associated with a worse outcome in HGSOC patients, and it might be used as a prognostic biomarker of cancer recurrence [128].

### 6.6. Low-Density Lipoprotein (LDL) Receptor-Related Protein 2 Binding Protein (LRP2BP)

According to the Human Protein Atlas, *LRP2BP* is equally expressed in all anatomical sites containing ciliated cells. There are no PubMed manuscripts reporting *LRP2BP*’s involvement in ciliary function or ciliogenesis. *LRP2BP* was originally cloned from human fetal brain; it contains four ankyrin repeat domains, which suggests a role in protein–protein interaction, and it also contains two casein kinase II phosphorylation sites and three protein kinase C phosphorylation sites [129]. Despite these predictions, there is no direct evidence of *LRP2BP* function. Indirect evidences associate *LRP2BP* with several diseases such as osteoporosis in an ovariectomized mouse model [130], intellectual disability [131], and atherosclerosis [132].

### 6.7. Leucine-Rich Repeat Containing 46 (LRRC46)

According to the Human Protein Atlas, *LRRC46* is equally expressed in all anatomical sites containing ciliated cells. After reviewing the current literature, we could find only one publication mentioning *LRRC46*, which reports the complete co-segregation of an *LRRC46* single-nucleotide variation (i.e., rs145648581) with prostate cancer (PCa) in one family with hereditary PCa [133]. However, the authors did not provide any hints on *LRRC46* function which could explain the increased risk for PCa. Interestingly, according to GeneCards [134], *LRRC6* is an important paralog of *LRRC46. LRRC6* is a gene that, when mutated, causes primary ciliary diskinesia. Indeed, cells with homozygous mutations of *LRRC6* do not have dynein arms in the axoneme of motile cilia of the respiratory tract or in the flagellum of spermatozoa [135]. LRRC6 interacts with other proteins of the dynein arms (e.g., Reptin/Ruvbl2 or ZMYND10), and, based on sequence similarities with *LRRC46*, we could expect a similar function for the latter.

### 6.8. Membrane-Associated RING-CH-Type Finger 10 (MARCH10)

According to the Human Protein Atlas, *MARCH10* is expressed in the female and male genital tract, and at least 10-fold less in the brain and airways. There is only one publication concerning *MARCH10*, which describes the cloning of the gene from rat testis and the characterization of *MARCH10* expression in developing spermatids, but not in epididymal spermatozoa [136]. *MARCH10* is transcribed in two different isoforms: MARCH10a (90 kDa) and MARCH10b (30 kDa). The first encodes for a RING finger protein with E3 ubiquitin ligase activity, while the second encodes a shorter isoform missing the RING finger domain. MARCH10a interacts with microtubules, and its E3 ubiquitin ligase activity is dependent on microtubule interaction [136].

### 6.9. Radial Spoke Head 10 Homolog B2 (RSPH10B2)

After reviewing the current literature, we could not find any publications concerning *RSPH10B2*. According to the Human Protein Atlas, *RSPH10B2* is expressed in the female and male genital tract, and at least 10-fold less in the brain and airways. Although the function of *RSPH10B2* is unknown, according to its protein sequence, *RSPH10B2* is part of the radial spoke in flagella and motile cilia [134,137]. The radial spoke is a protein complex that connects the nine MTDs to the CP of the 9 + 2 structure of motile cilia and flagella [138]. The radial spoke transfers the wave of ciliary beat between the CP and the MTDs through the dyneins. Radial spokes have a “T”-shaped structure, with the stalk interacting with the MTD and the orthogonal head interacting with the CP [137]. The loss of radial spoke head (RSPH) proteins (e.g., RSPH1, 3, 4a, and 9) impairs the ciliary or the flagellar beating, and causes primary cilia dyskinesia [139,140]. When mutated, RSPH proteins (e.g., RSPH4a) confer the motile cilia (9 + 2 structure) a clockwise rotation, instead of a typical planar beating similar to the node cilia (9 + 0 structure) in the embryo [141].

### 6.10. Sperm-Associated Antigen 6 (SPAG6)

According to the Human Protein Atlas, *SPAG6* is expressed in the female and male genital tract, and at least 10-fold less in the brain and upper airways. *SPAG6* was first identified as a novel human sperm antigen involved in male infertility [142]. Subsequent publications reported that SPAG6 is part of the central apparatus of the axoneme of motile cilia or flagella, and that it presumably controls flagellar or ciliary beat by interacting with SPAG16 and SPAG17 [143,144]. A knockout mouse model of *SPAG6* prematurely died due to hydrocephalus, and both male and female surviving animals showed infertility. These phenotypes can be correlated with impairment of ciliary activity in ependymal cells, in the flagella of male germinal cells, or in ciliary cells of the female oviduct, respectively [145,146]. *SPAG6*, *16*, and *17* promoters present a putative binding site of transcription factors involved in spermatogenesis (CREB/CREM, SOX17, and SPZ1) as well as ciliogenesis (FOXJ1) [144]. Within its own genomic interval, *SPAG6* is the only gene to be differentially expressed between FTE and HGSOC samples (Supplementary Table S3). *SPAG6* is also differentially expressed in several tumors (e.g., testicular germ-cell tumors, hematological malignancies, and breast and lung cancer) [147,148,149,150]. Interestingly, because *SPAG6* is an antigen of autoimmune male infertility and is expressed in several tumors, Silina et al. proposed its use as an antigen for anti-cancer immunotherapy [148].

### 6.11. Serine/Threonine Kinase 33 (STK33)

According to the Human Protein Atlas, *STK33* is equally expressed in all anatomical sites containing ciliated cells. Out of the 10 genes expressed in ciliated cells but not in serous cells of FTE, *STK33* is the only one also expressed in tissues that do not contain ciliated cells [151]. *STK33* is located within the chromosomal 11p15 region, which is associated with predisposition to various tumors [152]. An *STK33* knockout (KO) mouse was generated by removing exon 7, thus generating a truncated protein lacking the kinase domain. This mouse model was viable; however, it showed profound abnormalities in spermatogenesis, but not in ciliated cells. In fact, in this KO model, *STK33* expression was not lost in ciliated cells of the lung and oviduct that naturally express a spliced variant missing exon 7 and exon 8. Therefore, it appears that the STK33 kinase domain is not necessary in ciliated cells [153]. Among the 12 candidates, *STK33* is certainly the gene most studied in cancer, which is probably the case because Scholl et al. reported that *STK33* is indispensable for the survival of several *KRAS*-mutant tumor cells due to *STK33*-dependent suppression of mitochondrial apoptosis [154]; however, this same finding was not confirmed by other independent groups [155,156]. There are several publications that describe an inconsistent prognostic role of *STK33* in different tumors [157,158,159,160,161]. Quite interestingly, different publications or databases report inconsistent subcellular localization of STK33. For instance, the HPA reports that STK33 is localized mainly in the nucleus of tumor cell lines cultured in vitro, but, in tissue sections (either healthy or tumor), STK33 is mainly cytoplasmic. At the same time, it was reported that, in liver and pancreatic cancer, STK33 can localize both in the nucleus and in the cytoplasm, and that nuclear localization is associated with a poorer outcome [160,161]. A possible explanation of these apparent discrepancies is that STK33 subcellular localization is regulated by hypoxia, which favors STK33 nuclear translocation [160]. STK33 function in the nucleus is yet to be described.

### 6.12. Tubulin Polymerization-Promoting Protein (TPPP)

According to the Human Protein Atlas, *TPPP* is mostly enriched in the brain, where it was originally cloned [162], and it is also associated with Parkinson’s disease [163,164]. Based on HPA IHC data, in addition to brain localization, *TPPP* is present in pancreatic Langerhans islets, in FTE, and in the bronchus. The TPPP protein binds tubulin, favoring its polymerization [162]; this function is potentiated by TPPP dimerization, which is regulated by TPPP itself and GTP concentration. TPPP increases tubulin acetylation levels by interacting with HDAC6 and inhibiting its deacetylase activity. Despite having found *TPPP* expressed in ciliated cells, there is only one publication linking *TPPP* and cilia [165]; however, there are no insights into the role of TPPP in ciliated cells. Likewise, *TPPP* was found to be associated with several cancers [96,166,167,168]; however, no insights into its role in cancer were described either. Most likely, TPPP’s role in ciliated cells and cancer cells relies on TPPP’s regulation of microtubule dynamics; at the same time, we cannot exclude different TPPP functions in these cells, seeing as TPPP is a neomorphic moonlighting protein (i.e., a protein that can switch from a normal to a pathological function according to its protein partners and different conditions) [169].

## 7. Conclusions and Future Perspectives

Herein, we described the main characteristics of HGSOC and its most frequent risk factors (i.e., reproductive and genetic factors). In an attempt to identify novel candidate genes associated with EOC, we discovered that several of these genes are expressed in the ciliated cells of the FTE, and are responsible for regulating cilia motility. This finding prompted us to review the current knowledge surrounding the hypothesis that the malfunction of ciliated cells, despite not being the cell of origin of HGSOC, might increase the genotoxic stress environment after ovulation through an impaired follicular fluid clearance, thus preparing the soil for HGSOC onset.

To date, there are no experimental data directly supporting this hypothesis. Ideal strategies to study this hypothesis could include the modulation of gene expression of the genes regulating CBF (e.g., using short hairpin RNAs (shRNAs) or CRISPRs) in either KO mouse models [145,146,153] or in vitro models [82,83], in an effort to superimpose genotoxic stress (e.g., due to superovulation or using follicular fluid), and finally, to evaluate insurgence and latency of DNA damage signs according to References [50,82].

*CEP72* and *HOXB3* are expressed both in serous and ciliated cells (Figure 1); this evidence might suggest that other genes among those expressed in motile cilia could be present at much lower levels in fallopian serous epithelial cells. Proteins localized in motile cilia typically interact or regulate tubulin polymerization, and therefore, may also regulate other processes involving microtubules (e.g., cell morphology, motility, and mitosis). For instance, CCDC170 controls Golgi localization and the migration of cells [87]. *Spag6*-knockout murine embryonic fibroblasts had a different morphology, decreased motility, and more than two centrosomes, and were more sensitive to paclitaxel compared to their wild-type counterpart [170]. At the same time, proteins expressed in motile cilia may also be expressed in the primary cilium. The primary cilium is a single non-motile cilium, which is present in all human growth-arrested cells [171]. Primary cilia do not share the same architecture as motile cilia; instead, they contain a 9 + 0 structure, missing the central pair of microtubules. The primary cilium’s function seems to mainly involve the regulation of signaling transduction [171]. Despite differences in the microtubule architecture (9 + 2 versus 9 + 0), the protein compositions of motile and primary cilia are quite similar [172], and FTE serous cells have primary cilia [173]. Interestingly, primary cilia syndromes are characterized by an abnormal activation of the DNA damage response [94,174,175], which resembles one characteristic of HGSOC [7]. The hypothesis that the 12 genes, which we described herein to be expressed in ciliated cells, may also have a role in serous cells, and therefore, in HGSOC cells led us to explore the impact of these genes on HGSOC prognosis. We investigated these 12 genes using the Kaplan Meier-plotter website for ovarian cancer [176]; however, we could not find any consistent results, as described in the Supplementary Materials (Supplementary Table S7, Supplementary Figure S1 and Figure S2).

In conclusion, we reviewed the up-to-date findings concerning EOC/HGSOC predisposition, and our findings offer a novel perspective on the initial mechanisms involved in EOC/HGSOC, which can foster experimental research on the impact of FTE ciliated-cell clearance of oxidative stress.

## Figures and Tables

**Table 1:** List of candidate genes.

GWAS_Block	Coordinate_GWAS_Block	Gene ^a^	HPA Validation ^b^	HPA Localization	Subcellular Localization
GWAS_EOC_54	chr9:135138764-137155444	*AK8*	Enhanced	Ambiguous	NA
GWAS_EOC_52	chr9:15913285-17915021	*BNC2*	Approved	Ambiguous	NA
GWAS_EOC_25	chr19:16389703-40732752	*BST2*	Approved	Ambiguous	NA
GWAS_EOC_30	chr20:56330568-58330569	*C20orf85*	Enhanced	Ciliated cells	Brush border
GWAS_EOC_46	chr6:150405376-152405377	*CCDC170*	Enhanced	Ciliated cells	Brush border
GWAS_EOC_52	chr9:15913285-17915021	*CCDC171*	Uncertain	Ambiguous	NA
GWAS_EOC_12	chr11:85642871-87642872	*CCDC81*	Uncertain	Ambiguous	NA
GWAS_EOC_38	chr5:279789-2279790	*CEP72*	Approved	Serous and ciliated cells	NA
GWAS_EOC_20	chr16:83537526-85537527	*DNAAF1*	Enhanced	Ciliated cells	Cytoplasm and brush border
GWAS_EOC_5	chr1:243240447-245240448	*EFCAB2*	Approved	Ambiguous	NA
GWAS_EOC_42	chr5:174418048-176418049	*FAM153B*	Uncertain	Ambiguous	NA
GWAS_EOC_34	chr3:155397748-157435952	*GMPS*	NA	Not available	NA
GWAS_EOC_23	chr17:42516401-47500673	*HOXB3*	Approved	Serous and ciliated cells	NA
GWAS_EOC_26	chr19:38732751-40732752	*KCNK6*	Uncertain	Ambiguous	NA
GWAS_EOC_16	chr14:41173640-43173641	*LRFN5*	Uncertain	Not detected	NA
GWAS_EOC_37	chr4:184470585-186470586	*LRP2BP*	Approved	Ciliated cells	Brush border
GWAS_EOC_23	chr17:42516401-47500673	*LRRC46*	Enhanced	Ciliated cells	Nucleus, cytoplasm and brush border
GWAS_EOC_24	chr17:58880645-61480968	*MARCH10*	Enhanced	Ciliated cells	Nucleus and blefaroplast
GWAS_EOC_11	chr11:35386754-37386755	*PAMR1*	NA	Not available	NA
GWAS_EOC_35	chr4:118949959-120949960	*PDE5A*	Approved	Ambiguous	NA
GWAS_EOC_35	chr4:118949959-120949960	*PRSS12*	Uncertain	Ambiguous	NA
GWAS_EOC_48	chr7:6108187-8108188	*RSPH10B2*	Supported	Ciliated cells (not all)	Cytoplasm and brush border
GWAS_EOC_6	chr10:20827795-22915619	*SPAG6*	Enhanced	Ciliated cells	Cytoplasm and brush border
GWAS_EOC_8	chr11:7404500-9404501	*STK33*	Uncertain	Ciliated cells (not all)	Cytoplasm
GWAS_EOC_34	chr3:155397748-157435952	*TIPARP*	Uncertain	Ambiguous	NA
GWAS_EOC_11	chr11:35386754-37386755	*TNXB*	NA	Not available	NA
GWAS_EOC_38	chr5:279789-2279790	*TPPP*	Enhanced	Ciliated cells (not all)	Cytoplasm and brush border
GWAS_EOC_1	chr1:21415409-23490724	*WNT4*	Uncertain	Serous	NA

^a^ Colors indicate whether gene expressions are increased (green) or decreased (red) in one (light-green or red) or in two (dark-green or red) gene expression datasets (GSE69428 and GSE10971) by comparing high-grade serous epithelial ovarian cancers (HGSOC) and micro-dissected fallopian tube epithelia. ^b^ According to the Human Protein Atlas (HPA) antibody tissue validation. Supported: consistency with RNA sequencing (RNA-seq) and/or protein/gene characterization data. Approved: consistency with RNA-seq data in combination with inconsistency with, or lack of, protein/gene characterization data. Alternatively, consistency with protein/gene characterization data in combination with inconsistency with RNA-seq data. Uncertain: inconsistency with, or lack of, RNA-seq and/or protein/gene characterization data. Enhanced: consistency with the staining pattern using two single-target independent antibodies with non-overlapping epitopes. The spatial localization of the staining pattern generated by immunohistochemistry using the two antibodies was compared in 44 different normal tissues [64]. NA: not available

## Supplementary Materials

Supplementary materials can be found at http://www.mdpi.com/1422-0067/19/9/2512/s1.

## Abbreviations

HGSOC	High Grade Serous Epithelial Ovarian Cancer
EOC	Epithelial Ovarian Cancer
