# Supplementary material for: Exploring the Role of Fallopian Ciliated Cells in the Pathogenesis of High-Grade Serous Ovarian Cancer
Source: Int J Mol Sci. 2018 Aug 24;19(9):2512. doi: 10.3390/ijms19092512 (PMC6163198; doi:10.3390/ijms19092512)
Supplement: Supplementary file 1 [file ijms-19-02512-s001.zip › Coan&al._SupplementaryMaterial.docx]

**Supplementary Material**

Microarray gene expression analysis

GSE69428 and GSE10971 were downloaded from GEO Dataset (https://www.ncbi.nlm.nih.gov/gds/) and analyzed with R studio according to the attached pipeline (RStudio_analysis.txt)

Relationship between the expression of the candidate genes and the patient outcome

To investigate whether the expression of the candidate genes linked with EOC predisposition correlates with a different clinical outcome, we interrogated the Kaplan Meier-plotter database [178].

We evaluated the progression free survival (PFS) and the overall survival (OS) of OvCa patients with a serous histology and grade 2 or 3. Patients were grouped using the auto selection best cutoff method. For each gene analyzed, we used the best probe according to the Jet Set status, except for *RSPH10B2* that has only a probe classified as bad.

Data obtained by this analysis are reported in Table S7 and in Figure S1 and S2.

None of the candidate genes seem to correlate with a clinical outcome, but since we suppose these genes have a role in OvCa predisposition, they could be involved only in the first stage of the disease and do not interfere with the prognosis.

**Table S7.** PFS and OS analysis for the 12 candidate genes. In bold significant (*p* < 0,05) probe sets.

| **Gene** | **Probe** | **PFS HR** | **PFS logrank**  **P** | **OS HR** | **OS logrank P** |
| --- | --- | --- | --- | --- | --- |
| *C20orf85* | 229542_at | 1.15 (0.91 − 1.45) | 0.23 | **0.77 (0.61 − 0.98)** | **0.033** |
| *CCDC170* | 220581_at | **1.18 (1.02 − 1.37)** | **0.031** | **0.81 (0.69 − 0.94)** | **0.0071** |
| *CEP72* | 219531_at | **1.21 (1.04 − 1.4)** | **0.013** | 1.15 (0.96 − 1.38) | 0.12 |
| *DNAAF1* | 222068_s_at | 0.88 (0.76 − 1.02) | 0.1 | 0.89 (0.74 − 1.06) | 0.19 |
| *HOXB3* | 228904_at | **0.76 (0.6 − 0.98)** | **0.03** | 1.22 (0.95 − 1.55) | 0.12 |
| *LRP2BP* | 207797_s_at | **1.38 (1.17 − 1.62)** | **0.00012** | 0.88 (0.75 − 1.03) | 0.11 |
| *LRRC46* | 230600_at | 0.83 (0.67 − 1.03) | 0.083 | 0.87 (0.69 − 1.09) | 0.22 |
| *MARCH10* | 230824_at | 0.85 (0.68 − 1.07) | 0.17 | 1.15 (0.87 − 1.52) | 0.32 |
| *RSPH10B2* | 1557417_s_at | 0.83 (0.65 − 1.05) | 0.11 | 0.78 (0.6 − 1.01) | 0.056 |
| *SPAG6* | 210033_s_at | 1.16 (0.99 − 1.36) | 0.065 | 1.1 (0.94 − 1.28) | 0.25 |
| *STK33* | 228035_at | 0.86 (0.69 − 1.07) | 0.19 | 0.88 (0.7 − 1.1) | 0.26 |
| *TPP* | 230104_s_at | **1.29 (1.02 − 1.63)** | **0.031** | 1.24 (0.98 − 1.59) | 0.078 |


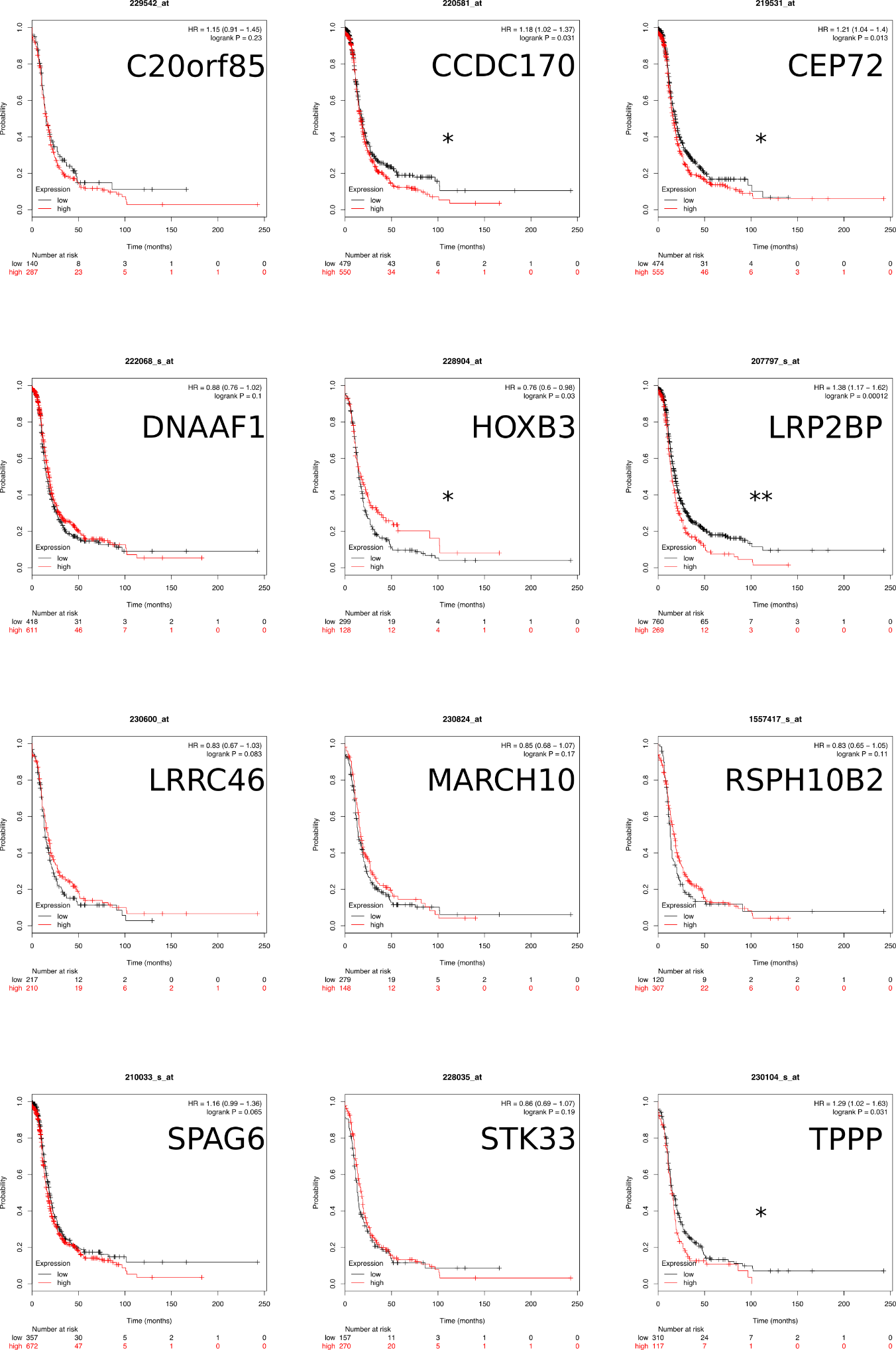


**Figure S1.** PFS graphs for the 12 candidate genes. Differences were considered significant at logrank *p* < 0.05 and < 0.01 and labeled accordingly (* or **, respectively).


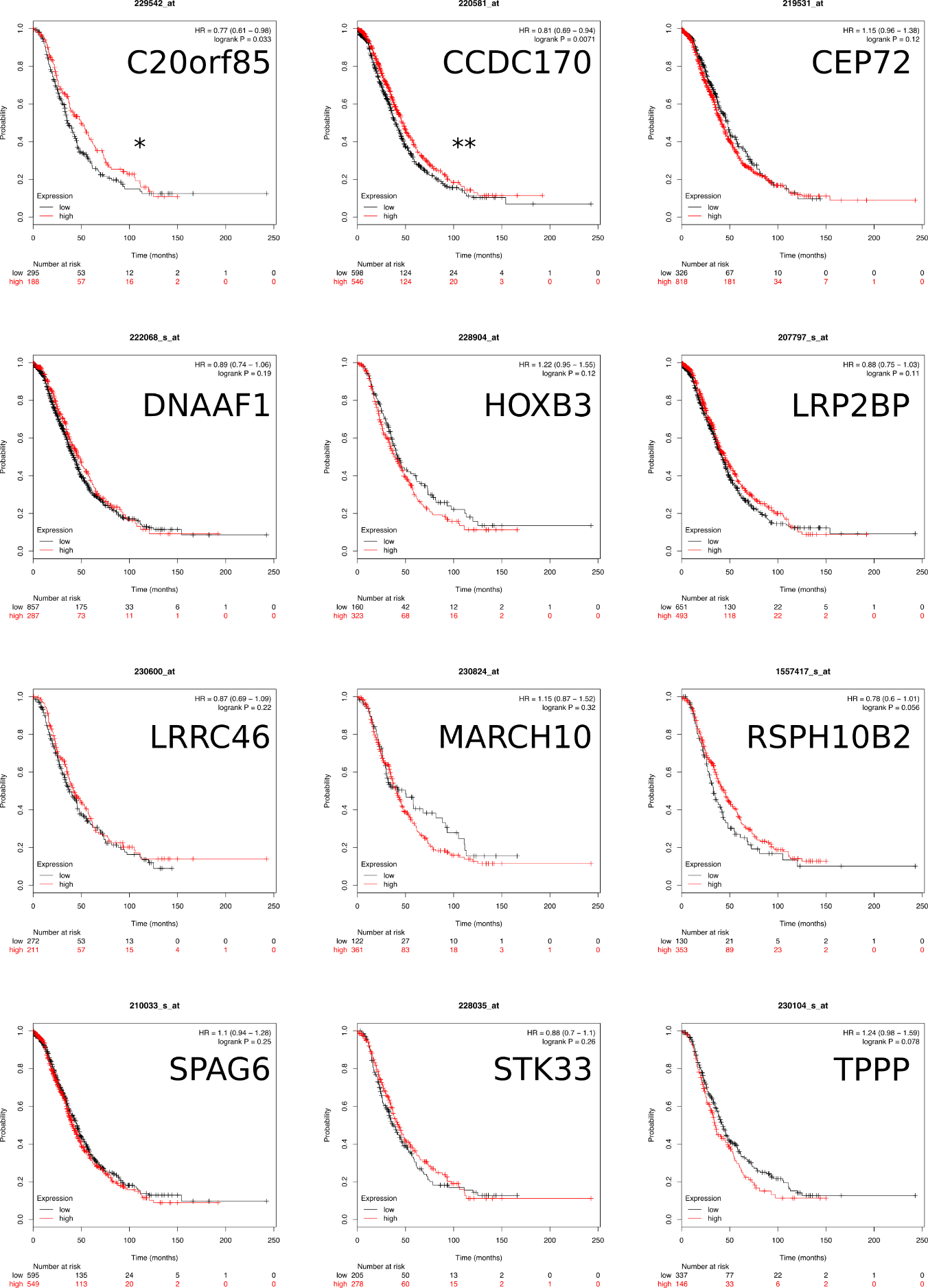


**Figure S2.** OS graphs for the 12 candidate genes. Differences were considered significant at logrank *p* < 0.05 and < 0.01 and labeled accordingly (* or **, respectively).
